# Optimal risk stratification and therapeutic strategy for acute myocardial infarction

**DOI:** 10.1002/clc.23645

**Published:** 2021-05-20

**Authors:** Teruhiko Imamura

**Affiliations:** ^1^ Second Department of Internal Medicine University of Toyama Toyama Japan

To the Editor

Given recent improvements in medical management, risk models for acute myocardial infarction are required to be updated. Wang et al. proposed a novel scoring method to predict short‐term mortality in patients with acute myocardial infarction.^1^ Several concerns have been raised.

In their study, the GRACE score was used as a comparator.^1^ In the real‐world practice, several other scorings are utilized, including TIMI risk score, PURSUIT risk score, and NCDR‐ACTION registry.^2^ Clinical implications would be further enhanced when the risk predictability of their novel score is compared with these other scorings.

Several parameters such as blood glucose and pulmonary artery systolic pressure included in their risk score are modifiable.^1^ The next concern is whether mortality would improve when these parameters are ameliorated by the aggressive intervention. It would be of great interest to investigate the prognostic impact of an improvement in the total score during the index hospitalization (between admission and discharge) in their cohort.

The prognostic impact of several parameters such as blood glucose might be “U‐shape” instead of linear, unlike their hypothesis.^1^ The cubic spline model might be more suitable to represent the accurate association between these parameters and mortality.

## Data Availability

There is no data utilized in this study.
